# From Environmental Exposure to Intervertebral Disc Degeneration: First Evidence of Pro-Degenerative Effects of Polyamide 6 Microplastics

**DOI:** 10.3390/biomedicines14061261

**Published:** 2026-05-31

**Authors:** Yong Sun, Xindi Bian, Yuchen Wang, Yizhi Zhang, Kun Wang, Shijie Chen, Lei Huang, Jizhe Peng, Zhaoxi Wang, Xuewen Kang

**Affiliations:** 1 Department of Orthopedics, Lanzhou University Second Hospital, Lanzhou 730030, China; suny2024@lzu.edu.cn (Y.S.); zhyizhi2023@lzu.edu.cn (Y.Z.); 19861172615@163.com (K.W.); chenshj2023@lzu.edu.cn (S.C.); 18530126250@163.com (L.H.); wangzhx2024@lzu.edu.cn (Z.W.); 2 Second Clinical Medical College, Lanzhou University (Chengguan Campus), Lanzhou 730030, China; bianxd2023@lzu.edu.cn (X.B.); wyuchen2024@lzu.edu.cn (Y.W.); pengjzh2025@lzu.edu.cn (J.P.); 3 Orthopaedics Key Laboratory of Gansu Province, The Second Hospital of Lanzhou University, Lanzhou 730030, China

**Keywords:** Polyamide 6 microplastics, intervertebral disc degeneration, GEO data mining, network toxicology, molecular docking

## Abstract

**Background:** Polyamide 6 microplastics (PA6-MPs), as emerging environmental pollutants, have attracted increasing attention due to their potential health risks. Their accumulation in human intervertebral disc tissue (86.4 particles/g) suggests a possible role in intervertebral disc degeneration (IVDD). However, direct evidence and mechanistic understanding remain limited. This study aimed to investigate the association between PA6-MPs exposure and IVDD, based on the hypothesis that PA6-MPs promote IVDD progression by targeting key regulatory molecules and disrupting cellular homeostasis. **Methods:** Potential PA6-related targets were predicted using multiple public databases, and IVDD-related differentially expressed genes were obtained from the GEO database. Overlapping targets were identified and analyzed through protein–protein interaction (PPI) network construction, Gene Ontology (GO), and Kyoto Encyclopedia of Genes and Genomes (KEGG) enrichment analyses to screen core targets and pathways. Molecular docking was performed to evaluate PA6–protein binding. In vitro validation was conducted using primary human nucleus pulposus cells exposed to PA6-MPs, with cell viability, proliferation, and phenotypic changes assessed by CCK-8, EdU, live/dead staining, and immunofluorescence (IF). **Results:** A total of 222 PA6-related targets and 1035 IVDD-associated genes were identified, yielding 10 overlapping targets. Four core targets, including NR3C1 and HDAC1, were selected. Molecular docking and experiments demonstrated stable binding and concentration-dependent inhibition of cell viability and proliferation. **Conclusion:** PA6-MPs may accelerate IVDD progression in a concentration-dependent manner by targeting key molecules and perturbing inflammatory homeostasis. These findings link environmental exposure to IVDD and provide a basis for future risk assessment and targeted intervention strategies.

## 1. Introduction

Microplastics (MPs) are plastic particles ranging in size from 1 μm to 5 mm, primarily formed through the degradation of plastic waste in the environment via physical abrasion, chemical oxidation, and biological activity [1]. These particles originate from diverse sources, including the washing of synthetic textiles, the breakdown of plastic products, and tire wear [2], and they have become ubiquitous in daily life. Presently, MP pollution has emerged as a critical global environmental issue. The ocean, a major hotspot for plastic contamination, receives an estimated 11 million tons of plastic waste annually [3]. The distribution of MPs is extensive, spanning from the Pacific Ocean to the Antarctic region and affecting over 100 marine species [4]. In the atmosphere, MPs are widely distributed across urban, rural, and remote areas, including glaciers and the Qinghai-Tibet Plateau [5], with their concentrations varying according to the level of urbanization [6]. It is estimated that land-based sources account for approximately 80% of the MPs found in the ocean, with particles entering the marine environment through pathways such as urban runoff, reaching concentrations as high as 8580 particles per liter in localized waters [7]. Given their widespread distribution, ability to migrate across environments, and high localized concentrations, MPs now represent a significant threat to global ecosystems.

Polyamide (PA) microplastics—commonly known as nylon—have garnered significant attention due to their close association with human life. Statistics reveal that, together with polyester, they account for over 90% of global synthetic clothing production. During use, wear, and laundering, PA garments continuously release PA-MPs into the environment [8]. This not only directly contaminates air and water bodies, but, due to their density (approximately 1.14 g/cm^3^), which is higher than that of water, PA-MPs are also more likely to accumulate within aquatic sediments, posing a long-term threat to aquatic ecosystems [9]. PA-MPs present in the environment primarily enter the human body through two main pathways: (1) respiratory inhalation of airborne suspended particles and (2) ingestion of particles via the food chain from contaminated water sources. Once in the body, PA-MPs can migrate through the bloodstream to various tissues, including the placenta, kidneys, and bone. Notably, studies have demonstrated that PA particles exhibit distinct specificity in bone tissue distribution. Specifically, they have been detected exclusively in intervertebral discs (IVD), with an abundance of 86.4 particles/g (based on a tissue weight of 1.1228 g) [10]. This observation suggests the potential presence of a unique bioaccumulation mechanism within IVD. However, the precise mechanisms behind this accumulation remain unclear, highlighting a critical research gap and underscoring the importance and value of investigating this phenomenon further.

In everyday life, PA primarily exists in two forms: PA6 and PA66. Among these, PA6 stands out due to its highest production volume and broadest range of applications, along with its unique physical and mechanical properties. This material has a melting point of approximately 205 °C and a glass transition temperature of 45 °C [11], which confer excellent heat resistance and dimensional stability. Its semi-crystalline structure provides an optimal balance between rigidity and toughness, while the polar amide bonds further enhance its mechanical strength and impact resistance. These characteristics make PA6 an ideal material for various sectors, including textiles, automotive manufacturing, and 3D printing [12]. However, precisely because of these properties, the MPs released by PA6 during prolonged use—resulting from friction and aging—are highly resistant to natural degradation. As a result, they persist and accumulate in the environment, contaminating ecosystems such as water bodies and soil. Of particular concern is the fact that PA6-MPs, due to their unique polarity and surface characteristics, exhibit an increased tendency to bind with proteins and trigger oxidative stress responses [13]. Notably, oxidative stress is recognized as one of the primary driving factors behind various degenerative diseases.

Intervertebral disc degeneration (IVDD) is a primary pathological cause of chronic low back pain and functional impairment, with its global incidence steadily rising. This increase imposes a substantial burden on both patient health and socioeconomic systems. The pathological hallmarks of IVDD include a reduction in the water content of the nucleus pulposus (NP), degradation of the extracellular matrix (ECM), structural damage to the annulus fibrosus (AF), and a loss of IVD height [14]. As the disease progresses, these changes can lead to further structural impairments, such as disc space narrowing, spinal stenosis, and segmental instability, thereby exacerbating spinal degenerative conditions [15]. The occurrence and progression of IVDD are synergistically regulated by multiple pathophysiological mechanisms: excessive reactive oxygen species (ROS) generated by oxidative stress can induce mitochondrial damage and energy metabolism imbalance, activating Caspase family and Bcl-2 family-mediated cell apoptosis [16]. This process is closely associated with monoamine oxidase B (MAOB)-regulated oxidative burst and nuclear receptor subfamily 3 group C member 1 (NR3C1)-mediated stress protection pathways [17,18]. Meanwhile, chronic inflammation participates throughout the entire process of IVDD, with massive release of pro-inflammatory cytokines such as tumor necrosis factor-alpha (TNF-α) and interleukin-1β (IL-1β), which activate nuclear factor kappa-B (NF-κB) and mitogen-activated protein kinase (MAPK) signaling pathways, accelerating extracellular matrix (ECM) degradation and inhibiting the repair capacity of intervertebral disc cells [19]. NR3C1 dysfunction further weakens anti-inflammatory capacity and amplifies inflammatory damage [20]. Furthermore, nucleus pulposus cell dysfunction manifests as reduced cell viability, limited proliferative capacity, and imbalanced autophagy levels [21], while aldose reductase (AKR1B1)-mediated metabolic disorders and oxidative stress also play important roles in this process, further exacerbating the local degenerative microenvironment [21]. Notably, histone deacetylase 1 (HDAC1), as a key epigenetic regulator, participates in the IVDD process by influencing inflammatory gene transcription and apoptotic pathways [22]. These molecular mechanisms collectively constitute the complex regulatory network underlying the occurrence and progression of IVDD.

In addition to genetic and mechanical factors, the role of environmental factors in the development of IVDD is receiving increasing attention. Recent studies have shown that PA are detectable exclusively within IVD tissues, but are absent from other bone tissues. This observation suggests the potential existence of a tissue-specific bioaccumulation mechanism. Regarding the underlying mechanisms of toxicity, current hypotheses suggest that the degradation products of PA may directly interfere with the normal metabolic processes of IVD cells [23]. PA6, the predominant form of PA present, is likely to interact with the IVD—potentially through pathways involving the induction of inflammatory responses and oxidative stress—thereby accelerating the degenerative process. Consequently, investigating the potential link between PA6-MPs and IVDD holds significant scientific importance for the prevention and treatment of this disease, particularly from the perspective of environmental exposure.

## 2. Methods

### 2.1. Collection of Polyamide 6 Targets

PA6 is an aliphatic polyamide with the molecular formula C_6_H_11_NO, consisting of carbon, hydrogen, nitrogen and oxygen. It has a linear molecular chain containing repeating amide groups, exhibiting strong polarity and hydrogen bonding, which confers stable physicochemical properties. Based on this structural information, potential targets of PA6 were retrieved from the ChEMBL (https://www.ebi.ac.uk/chembl/, accessed on 7 November 2025) database by restricting the species to Homo sapiens. The PA6 SMILES was further submitted to SwissTargetPrediction (http://www.swisstargetprediction.ch, accessed on 7 November 2025), SEA (https://sea.bkslab.org/, accessed on 7 November 2025), and TargetNet (http://targetnet.scbdd.com/, accessed on 7 November 2025) databases for additional target prediction, to identify potential binding proteins. All predicted targets were carefully verified for structural consistency. The target entries obtained from different databases were integrated, duplicates were removed. Finally, the consolidated targets were compiled to construct the potential target library of PA6.

### 2.2. Screening of Disease Targets Associated with Intervertebral Disc Degeneration

To identify genes closely associated with IVDD, we retrieved the GSE23130 dataset from the GEO database (https://www.ncbi.nlm.nih.gov/geo, accessed on 13 November 2025), which includes 15 healthy control samples and 8 IVDD patient samples. Differential gene expression analysis was performed using R software (version 4.1.2, R Core Team, 2021; https://www.R-project.org/, accessed on 13 November 2025), applying screening criteria of *p* < 0.05 and |log2FC| ≥ 0.5. The differentially expressed genes were visualized through volcano plots, heatmaps, Principal Component Analysis (PCA), and gene expression plots, thereby providing a clear and intuitive illustration of the significant changes in gene expression occurring in the disease state. These differentially expressed genes are considered potential disease targets for IVDD.

### 2.3. Construction of Protein-Protein Interaction Networks and Screening of Core Target Genes

To further elucidate the interaction network between PA6 and IVDD-related genes, the potential targets of PA6 were intersected with the differentially expressed genes associated with IVDD to identify common targets. These intersecting target genes were then imported into the STRING database (version 11.5, https://cn.string-db.org/, accessed on 25 November 2025) using the following parameters: species set to *Homo sapiens* and a minimum interaction score > 0.4. Subsequently, the CytoHubba plugin (version 0.1) within Cytoscape software (version 3.10.0, https://cytoscape.org/, accessed on 25 November 2025) was utilized to screen for core targets based on their Betweenness, Degree, and Closeness centrality values. In the resulting network visualization, the size and color intensity of the nodes intuitively reflect the degree values of the genes; a higher degree value generally indicates that a gene possesses more connections within the network and is therefore more likely to play a pivotal biological role. These core targets may play significant roles in the mechanisms by which PA6 influences IVDD.

### 2.4. Gene Ontology and Kyoto Encyclopedia of Genes and Genomes Enrichment Analysis

To gain a deeper understanding of the biological functions and involved signaling pathways of the intersecting targets, these targets were subjected to both Gene Ontology (GO) functional enrichment analysis and Kyoto Encyclopedia of Genes and Genomes (KEGG) pathway enrichment analysis using DAVID (version 6.8; https://davidbioinformatics.nih.gov/, accessed on 25 November 2025). For the GO enrichment analysis, a *p*-value threshold was applied as the filtering criterion, and a bubble chart was generated to visualize the results. Similarly, for the KEGG enrichment analysis, a *p*-value threshold was utilized to identify significantly enriched pathways, and a KEGG enrichment plot was generated to facilitate the visualization and analysis of these signaling pathways.

### 2.5. Molecular Docking Technology

Molecular docking was used to predict the interactions between PA6-MPs and core target proteins. PA6 is an aliphatic polyamide with the repeating unit –(CH_2_)_5_CONH– and molecular formula (C_6_H_11_NO)n; Four core hub genes were screened out via PPI network analysis, and their corresponding UniProt IDs were listed as follows: NR3C1 (1M2Z), MAOB (1GOS), HDAC1 (4BKX) and AKR1B1(1ABN). Binding energies were calculated using AutoDock (version4.2.6; https://autodock.scripps.edu/download-autodock4/, accessed on 5 December 2025), and the binding sites were visualized using PyMOL (version 2.5.2, Schrödinger, LLC; https://pymol.org/, accessed on 5 December 2025).

### 2.6. Experimental Validation

In this study, we conducted subsequent experimental validations using PA6-MPs from Siyeye Plastics (Shenzhen, China); primary human nucleus pulposus cells (H10936A) from Yaji Biotechnology (Shanghai, China); antibodies against NR3C1 and HDAC1 from Immunoway (Beijing, China); fluorescent secondary antibodies from Proteintech (Wuhan, China); a CCK-8 assay kit and a Calcein-AM/PI live/dead cell double-staining kit from Solarbio (Beijing, China); and an EdU kit from Beyotime (Shanghai, China).

#### 2.6.1. The Preparation of Polyamide 6 Microplastics Suspension

PA6-MP powder was added to complete culture medium supplemented with 10% fetal bovine serum (FBS), which was selected based on standard culture conditions commonly used for human nucleus pulposus cells to ensure stable cell viability and proliferation. The mixture was initially dispersed by vortex oscillation for 1 min, followed by water-bath sonication (40 kHz, 100 W, 10 min) to achieve sufficient dispersion of particles. The suspension was vortexed again immediately before use and promptly added to cell culture wells to minimize reaggregation caused by standing. Protein components in serum-supplemented medium can act as stabilizers and inhibit rapid agglomeration of particles to a certain extent.

In this study, the exposure concentrations of PA6-MPs were determined based on previous microplastic toxicological studies and our preliminary experimental results [24,25,26]. The selected dose gradient was designed with reference to commonly used concentration ranges and established grouping strategies in human cell models, thereby ensuring the rationality and reproducibility of the in vitro cytotoxicity analysis.

Combined with reported microplastic size data in human intervertebral disc and cartilage tissues [10], relevant conversion analysis confirmed that the actual microplastic exposure level in human local tissues was markedly higher than the in vitro concentration adopted in this study. Thus, our experimental concentration setting is conservative and physiologically relevant.

#### 2.6.2. CCK-8 Assay for Cell Viability

The CCK-8 assay was performed strictly according to the manufacturer’s instructions of the CCK-8 Cell Proliferation and Cytotoxicity Assay Kit (Solarbio, Beijing, China). Nucleus pulposus cells (NPs) were seeded into 96-well plates at a density of 5 × 10^3^ cells per well. Simultaneously, PA6-MP solutions at various concentrations (0, 5, 10, 20, 40,80 and 100 μg/mL) were prepared using DMEM/F-12 medium (Gibco, Grand Island, NE, USA) and added to the wells for co-culture with the NPs. Cells were cultured for 24 h, 48 h, and 72 h. Subsequently, 10 μL of CCK-8 reagent was added to each well, followed by incubation at 37 °C in a 5% CO_2_ incubator for 2 h in the dark. Subsequently, absorbance values at 450 nm were measured using a Bio Tek microplate reader (Winooski, VT, USA), and cell viability was evaluated based on the OD values.

#### 2.6.3. Live/Dead Cell Staining

For live/dead cell staining, a Live/Dead Cell Detection Kit (Solarbio, Beijing, China) was used. NPs were seeded into 6-well plates at a density of 2 × 10^5^ cells per well. The treatment medium for the experimental group was prepared by dissolving PA6-MPs in DMEM/F-12 medium (Gibco) to a final concentration of 50 μg/mL. The experimental group was cultured in this medium containing PA6-MPs, while the control group received an equal volume of standard culture medium. After 48 h of incubation, the cell culture supernatant of each group was discarded, and the cells were washed with pre-chilled PBS. A pre-prepared mixture of calcein-acetoxymethyl ester (Calcein-AM, Solarbio, Beijing, China) and propidium iodide (PI, Solarbio, Beijing, China) staining solution was added, followed by incubation at room temperature for 20 min in the dark. After incubation, the staining solution was removed and the cells were rinsed with PBS. Images were captured using an upright fluorescence microscope (Olympus, Tokyo, Japan). Live cells were stained green by Calcein-AM, while dead cells were stained red by PI.

#### 2.6.4. EdU Assay for Verifying Cell Proliferation Activity

The experimental and control groups were treated as described previously. After 24 h of incubation, EdU working solution prepared with an EdU Cell Proliferation Assay Kit (Beyotime, Shanghai, China) was added to each culture system at an appropriate ratio according to the manufacturer’s instructions, and cells were incubated at 37 °C in a 5% CO_2_ incubator for 4 h in the dark to label proliferative cells. Subsequently, the working solution was removed, and cells were processed through fixation, permeabilization and washing before being mounted with anti-fluorescence quenching mounting medium containing DAPI. Stained cells were observed and imaged using an Olympus upright fluorescence microscope (Olympus, Tokyo, Japan).

#### 2.6.5. Immunofluorescence Experiment

The experimental and control groups were subjected to the same treatments as described previously. After 24 h, the NPs were first fixed with 4% paraformaldehyde, permeabilized with 0.5% Triton X-100 (Beyotime, Shanghai, China), and then blocked with 5% bovine serum albumin (BSA). Subsequently, rabbit primary antibodies against NR3C1 and HDAC1 were diluted at a 1:200 ratio and incubated at 4 °C for 12 h. Following this, goat anti-rabbit secondary antibodies were incubated with the NPs at room temperature for 1 h. The samples were then stained with an anti-fluorescence quenching mounting medium (containing DAPI) (Beyotime, Shanghai, China), and finally, the cells were imaged using an Olympus upright fluorescence microscope (Olympus, Tokyo, Japan). Images were observed and captured under the fluorescence microscope at different magnifications.

### 2.7. Statistical Analysis

All data in this study were processed using IBM SPSS Statistics software (version 22.0, Windows 10 64-bit; IBM Corp., Armonk, NY, USA). Comparisons among multiple groups were performed using the Kruskal–Wallis test. If the results were statistically significant, a subsequent Dunn’s post-hoc test was conducted. Correlations between variables were assessed using Spearman’s rank correlation analysis. A *p*-value of <0.05 was considered statistically significant.

## 3. Results

### 3.1. Identification of Disease-Associated Differentially Expressed Genes

First, the data were normalized. Differential expression analysis comparing the normal and disease groups identified a total of 1035 differentially expressed genes, with 999 genes showing upregulated expression and 36 genes showing downregulated expression. These differentially expressed genes were then visualized using volcano plots, heatmaps, PCA, and violin plots.

**Figure 1:**
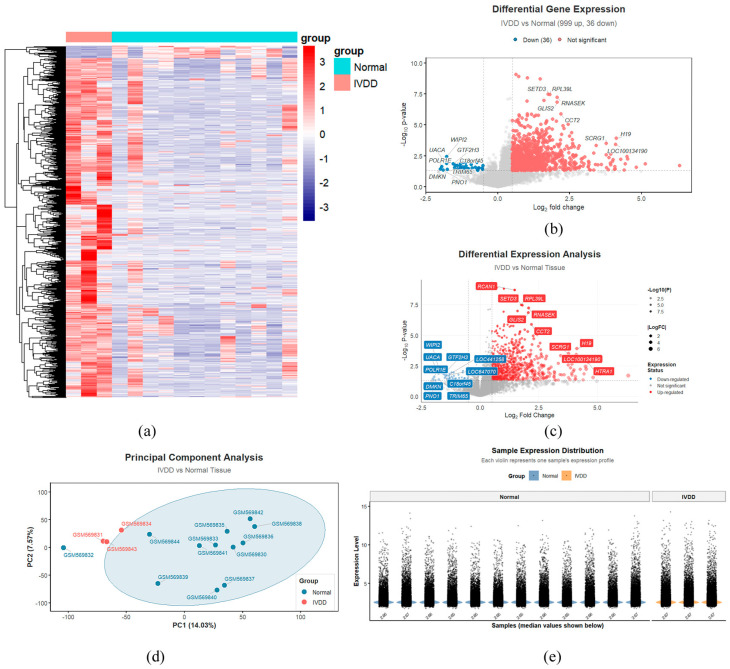
Differential gene analysis of the GSE23130 dataset. (**a**) A heatmap of the GSE23130 dataset illustrating the expression profiles of 1035 differentially expressed genes across groups. (**b**,**c**) Volcano plots of the GSE23130 dataset displaying the differential expression of all genes across groups; red indicates upregulation, and blue indicates downregulation, and grey indicates genes with no significant differential expression. (**d**) PCA plot, where blue circles represent Normal samples, red circles represent IVDD samples, and the blue ellipse represents the 95% confidence ellipse of sample distribution. (**e**) Expression distribution across samples, where black dots represent gene expression values and orange lines indicate the median expression level of each sample.

We generated a hierarchical clustering heatmap (Figure 1a) to further refine the differential gene expression analysis. The heatmap shows that the disease and control groups form two distinct clusters, indicating that the IVD tissues of patients with IVDD exhibit systematic and specific transcriptional alterations compared to those of healthy individuals. This finding not only validates the reliability of the data but also highlights the consistency of these differences.

To validate the differences between the normal and IVDD groups, a volcano plot (Figure 1b,c) and a sample expression plot (Figure 1e) were generated. The volcano plot shows that the majority of significantly differentially expressed genes are clustered on the right side, indicating that, compared to the normal group, genes in the IVDD group are significantly upregulated (represented by red dots). Using a threshold of *p* < 0.05, a total of 1035 differentially expressed genes were identified, including 999 upregulated genes and 36 downregulated genes. The sample expression plot further illustrates the differences in gene expression distribution between the two groups, suggesting that the core of the IVDD pathological process may involve the aberrant activation of numerous genes. This could potentially encompass processes such as inflammatory bursts, abnormal proliferation, or dedifferentiation.

A PCA plot (Figure 1d) was generated to further assess the differences in gene expression between the normal and IVDD sample groups. The two groups exhibited distinct separation within the principal component space, with no significant overlap between individual sample points. This indicates that the gene expression profiles associated with the IVDD state differ substantially from those of normal tissue, ruling out the confounding effects of individual variability and providing a solid data foundation for the current study.

Through differential gene expression analysis, this study clearly revealed significant and systematic differences in gene expression profiles between the IVDD sample group and the healthy control group. Various visualization methods—including heatmaps, volcano plots, and PCA—consistently demonstrated that the two groups were clearly distinguishable. This effectively ruled out the influence of significant individual variations, validating both the reliability of the data and the specificity of the disease state. These findings suggest that the pathological core of IVDD may involve the aberrant activation of numerous genes, closely linked to processes such as inflammatory bursts, abnormal cellular proliferation, or dedifferentiation, thereby providing a solid data foundation for subsequent mechanistic investigations.

### 3.2. Component-Target Map

**Figure 2:**
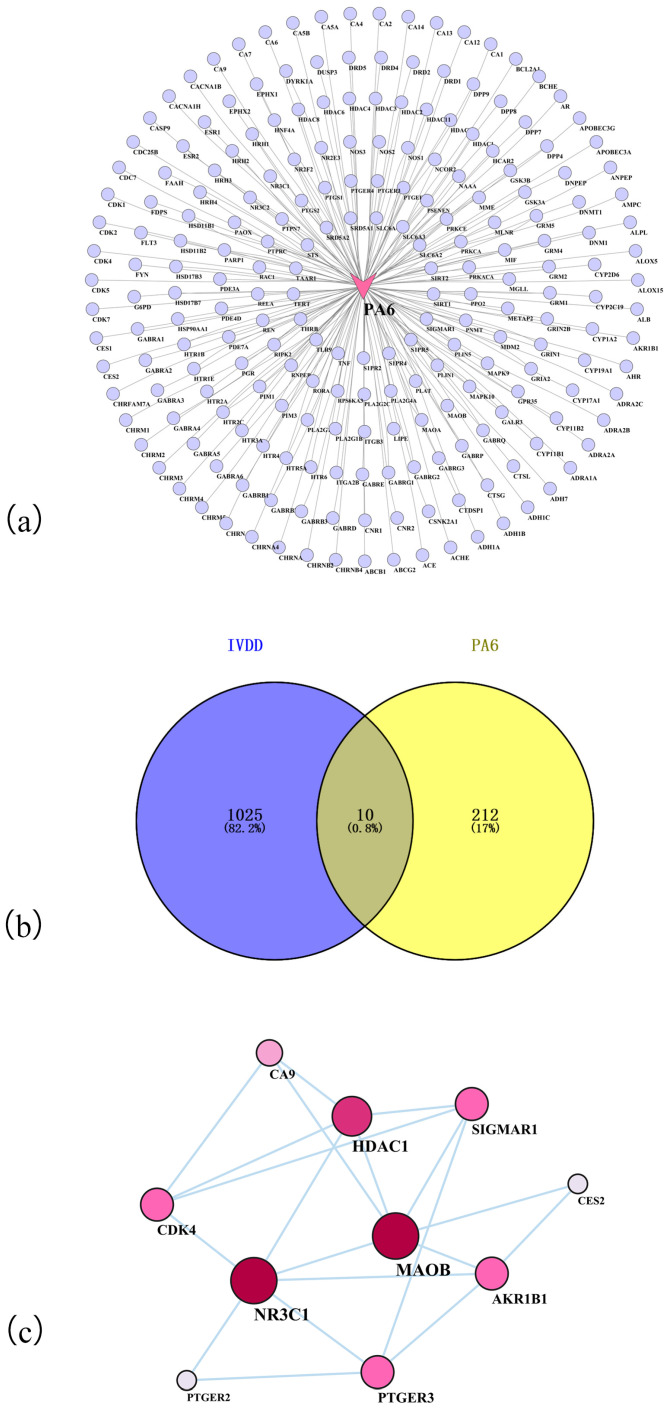
(**a**) PA6 Components–Targets Map. (**b**) Venn diagram showing the intersecting targets between PA6 and IVDD. (**c**) PPI Network and Component-Target-Disease Network.

To investigate the targets of PA6 action, a network analysis was conducted. We obtained the molecular structure of PA6, identified potential targets via database searches, performed cross-validation, merged data sources, removed duplicates, and verified structural consistency. This process yielded 222 potential targets of PA6. These targets were then imported into Cytoscape 3.10.0 for visualization, generating a PA6 component-target network diagram (Figure 2a) comprising 222 interaction edges. In this network, the PA6 node serves as the sole central hub, with all targets being directly regulated by it.

### 3.3. Identification of Polyamide 6—Induced Targets in Intervertebral Disc Degeneration

To investigate the potential molecular links between PA6 exposure and IVDD, the 222 identified PA6 targets were intersected with 1035 disease targets. After integration and deduplication, a total of 10 intersecting targets were identified as potential targets for PA6-MP-induced IVDD, and a Venn diagram was constructed (Figure 2b). These findings suggest that the 10 targets likely represent the critical link between PA6-MP exposure and IVDD, shedding light on the potential mechanisms through which PA6-MP exposure may contribute to IVDD progression and providing candidate genes for further investigation.

### 3.4. Construction of Protein–Protein Interaction Network and Screening of Active Ingredients

**Figure 3:**
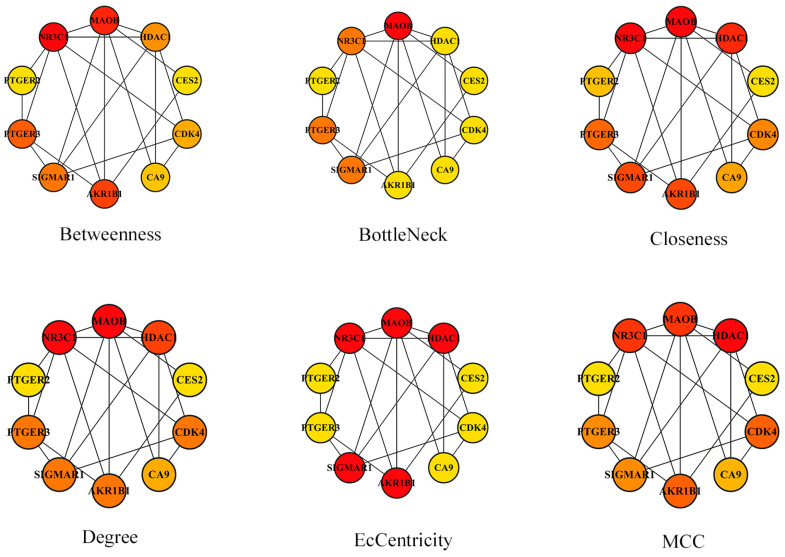
Core Target Screening Process—Corresponding to Betweenness, Bottleneck, Closeness, Degree, Eccentricity, and MCC, respectively.

To further identify key targets, the 10 intersecting targets obtained through screening were imported into the STRING database for protein-protein interaction (PPI) network analysis (Figure 2c). In this network, the nodes for NR3C1 and MAOB are the largest in size and darkest in color, indicating their high connectivity and involvement in multiple signaling pathways, such as those regulating inflammation, metabolism, and oxidative stress responses. Table 1 shows that NR3C1 and MAOB have the highest Degree and Betweenness values, signifying that they are central to the small network formed by these 10 targets. The six sub-figures in Figure 3 provide various perspectives on the core target proteins: NR3C1, MAOB, HDAC1, and AKR1B1. Together, these analyses highlight the critical roles these proteins play within the PPI network, suggesting that—as key targets—they likely contribute significantly to the progression of IVDD under PA6 exposure and may serve as promising therapeutic targets.

### 3.5. Gene Ontology Analysis Results for Shared Targets of Drug Treatments and Diseases

To investigate the potential biological processes or functions associated with the 10 candidate targets, enrichment analysis was performed using the DAVID database. This analysis generated a GO enrichment plot, where the y-axis represents the enriched terms and the x-axis represents the enrichment scores. The color of the data points transitions from blue to red, with red indicating a lower *p*-value, thus reflecting more significant enrichment.

**Figure 4:**
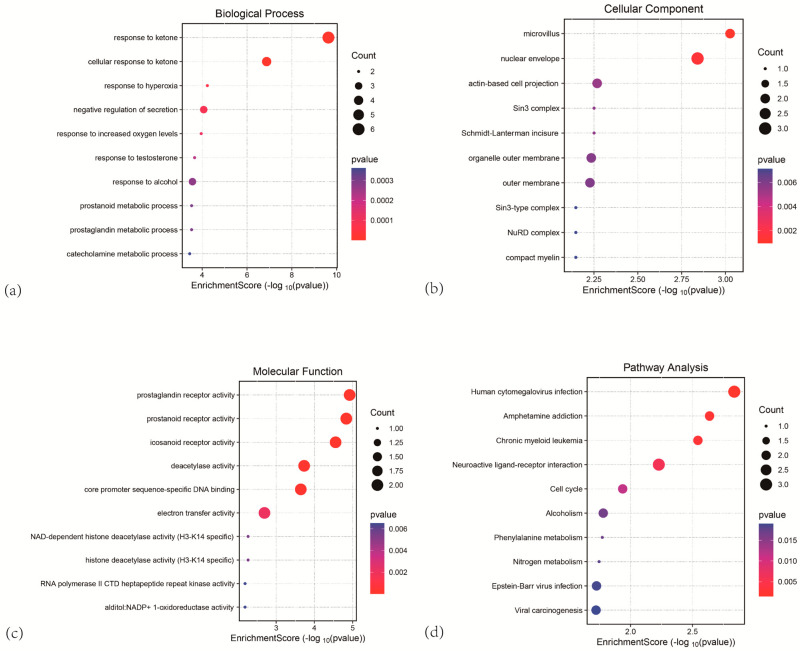
(**a**–**c**) GO Enrichment Analysis of Potential Targets. (**d**) Enrichment Analysis of Potential KEGG Targets.

The size of each point corresponds to the number of genes enriched within each specific term. The GO enrichment analysis elucidates gene functions across three dimensions: Biological Process (BP) (Figure 4a), Cellular Component (CC) (Figure 4b), and Molecular Function (MF) (Figure 4c). Analysis of the results reveals that, at the BP level, differentially expressed genes are significantly enriched in ketone-related stress response pathways. Notably, the “response to ketones” term shows the highest degree of enrichment (*p* < 1 × 10^−4^), suggesting that HDAC1 activity is likely suppressed by ketone bodies, with HDAC1 acting as a molecular bridge to indirectly influence NR3C1 function, thereby modulating inflammatory responses. Additionally, terms such as “response to hypoxia,” “negative regulation of secretion,” and “response to increased oxygen levels” are also enriched, implying that oxidative stress and metabolic stress may contribute to the cellular damage induced by PA6-MPs. At the CC level, differentially expressed genes are primarily localized to microvilli and the nuclear envelope (*p* < 0.002). As a critical interface between the cytoskeleton and intranuclear signal transduction, structural perturbations of the nuclear envelope further support the hypothesis that the nuclear translocation and functional regulation of core proteins, such as NR3C1, may be directly influenced by PA6-MPs. Moreover, terms like “actin-dependent cell projection,” “Sin3 complex,” and “outer membrane of organelle” are significantly enriched, indicating that PA6-MP exposure may disrupt the functional homeostasis of the cytoskeleton and membrane-associated structures. At the MF level, differentially expressed genes are highly enriched in inflammation-related receptor activities, specifically prostaglandin receptor activity, prostanoid receptor activity, and leukotriene receptor activity (*p* < 0.002). Additionally, terms such as “deacetylase activity,” “core promoter sequence-specific DNA binding,” and “electron transfer activity” are notably enriched, suggesting that the activation of inflammatory signaling pathways, epigenetic regulation, and energy metabolic disturbances represent the key molecular signatures through which PA6-MPs promote IVDD. These findings establish a link between the effects of PA6-MPs and epigenetic regulatory networks, suggesting that HDAC1-mediated histone deacetylation modifications may modulate inflammatory responses in IVD cells, thereby may contribute to IVDD progression by remodeling chromatin structure. Collectively, these results suggest that PA6-MPs may contribute to the pathological processes of IVDD by disrupting specific pathways, particularly those associated with NR3C1 and HDAC1.

### 3.6. Kyoto Encyclopedia of Genes and Genomes Metabolic Pathway Enrichment Analysis

To investigate how potential targets function synergistically within the biological system, and to identify the metabolic, signaling, and other biological pathways in which they may participate, a KEGG pathway enrichment analysis was performed. In the resulting visualization, the y-axis represents the various pathways, the bubble size corresponds to the number of genes, the x-axis indicates the enrichment score, and the color intensity reflects the −log(*p*-value), with the *p*-value serving as the criterion for statistical significance. This analysis revealed key biological processes that PA6-MPs may regulate in the context of IVDD. A total of 10 enriched signaling pathways were identified, ranked according to their *p*-values, and a KEGG enrichment plot was generated for visualization, as shown in Figure 4d.

The KEGG enrichment analysis diagram clearly illustrates the specific signaling pathways in which the differentially expressed genes are involved, indicating that these genes do not function in isolation. The results reveal significant enrichment of the differentially expressed genes across 10 signaling pathways. Notably, the “Human cytomegalovirus infection” pathway shows the highest degree of enrichment (*p* < 0.005), followed by the “Amphetamine addiction” and “Chronic myeloid leukemia” pathways. This suggests that stress associated with viral infections, neurotransmitter regulation, and hematopoiesis-related signaling pathways may contribute to the cellular damage induced by PA6-MPs. Additionally, the “Neuroactive ligand-receptor interaction” and “Cell cycle” pathways demonstrate significant enrichment (*p* < 0.01), implying that, given NR3C1’s role as a member of the nuclear receptor family, its ligand binding and signal transduction processes are likely directly regulated by PA6-MPs. Furthermore, pathways such as “Alcoholism,” “Phenylalanine metabolism,” “Nitrogen metabolism,” “Epstein-Barr virus infection,” and “Viral carcinogenesis” were also significantly enriched, suggesting that metabolic disturbances and virus-related oncogenic signaling pathways may synergistically contribute to the degenerative process. Considering the role of HDAC1 in histone modification and gene expression regulation, PA6-MPs may exert their effects by modulating these associated pathways, influencing cell cycle checkpoints and the transcriptional repression of inflammatory genes, and thus may potentially contribute to the pathological progression of IVDD. Collectively, these results suggest that exposure to PA6-MPs likely contributes to the progression of IVDD through the modulation of these relevant signaling pathways.

Based on the analysis of the charts above, we can outline the “molecular story” of how PA6-MPs influence IVDD:

Upon entering the body, PA6 may directly bind to and disrupt multiple key proteins, including NR3C1 and HDAC1. The core mechanism may potentially involve the induction of reactive oxygen species (ROS) burst mediated by factors including MAOB [27], which could affect the biological functions of key target proteins such as NR3C1. This disruption affects anti-inflammatory and stress homeostasis, induces metabolic dysregulation, interferes with cytoskeletal and intranuclear signaling, and compromises the antioxidant capacity of the ECM.

Downstream, the combined effects of uncontrolled inflammation, oxidative stress, disrupted cellular homeostasis, metabolic dysregulation, and altered neurotransmitter regulatory pathways activate massive transcriptional reprogramming within IVD tissues. This process impairs the structure and function of IVD cells, leading to apoptosis, senescence, oxidative stress, and ECM degradation, ultimately associating with IVDD-related cellular alterations.

Therefore, PA6 exposure may influence the development of IVDD through multiple mechanisms. GO analysis investigated the biological processes or functions in which the potential targets may be involved, while KEGG analysis explored the biological pathways—such as metabolism and signal transduction—in which these targets may participate within the organism. These findings provide a crucial reference for subsequent studies investigating the molecular mechanisms underlying PA6-induced IVDD.

### 3.7. Polyamide 6 Molecular Docking and Core Target Proteins in Intervertebral Disc Degeneration

**Figure 5:**
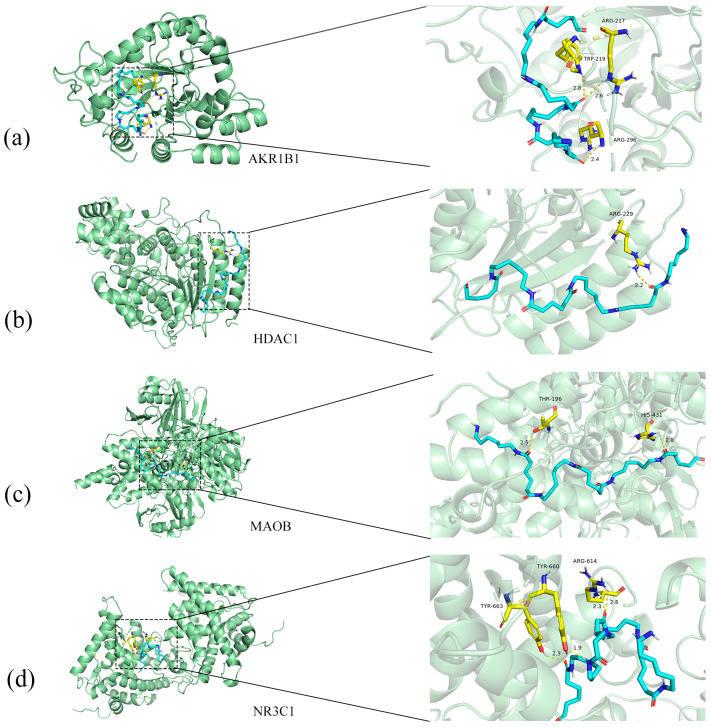
Molecular Docking Visualization Analysis: (**a**) AKR1B1; (**b**) HDAC1; (**c**) MAOB; (**d**) NR3C1. (Cyan: ligand; yellow: key interacting residues; light green: protein backbone; dashed lines: interactions with distances in Å.)

To investigate the potential interactions between PA6 and key regulatory targets associated with IVDD, we conducted a systematic molecular docking analysis (Figure 5). The study focused on four core target proteins: NR3C1, MAOB, HDAC1, and AKR1B1. Through computational simulations, we evaluated the binding modes and affinities of the PA6 molecule within the active sites of each target protein. The molecular docking results indicated that PA6 exhibits strong binding potential with all four core target proteins, with calculated binding energies consistently negative (all <0 kcal/mol). Lower binding energies generally indicate higher binding affinity and stability. From a computational perspective, these results support the hypothesis that PA6 may possess potential binding capacity with these targets, suggesting that they may be involved in the molecular mechanisms underlying PA6-induced IVDD.

Collectively, these bioinformatics analysis steps constitute a comprehensive framework for exploring the molecular mechanisms of PA6 in the treatment of IVDD—ranging from compound target prediction and the screening of disease-associated genes to the elucidation of core interaction networks and the identification of functional pathways—thereby providing a solid theoretical foundation and key target leads for subsequent experimental validation.

### 3.8. Polyamide 6 Microplastics Significantly Inhibit Nucleus Pulposus Proliferation and Interfere with Intracellular Transcriptional Regulation and Epigenetic Modifications

**Figure 6:**
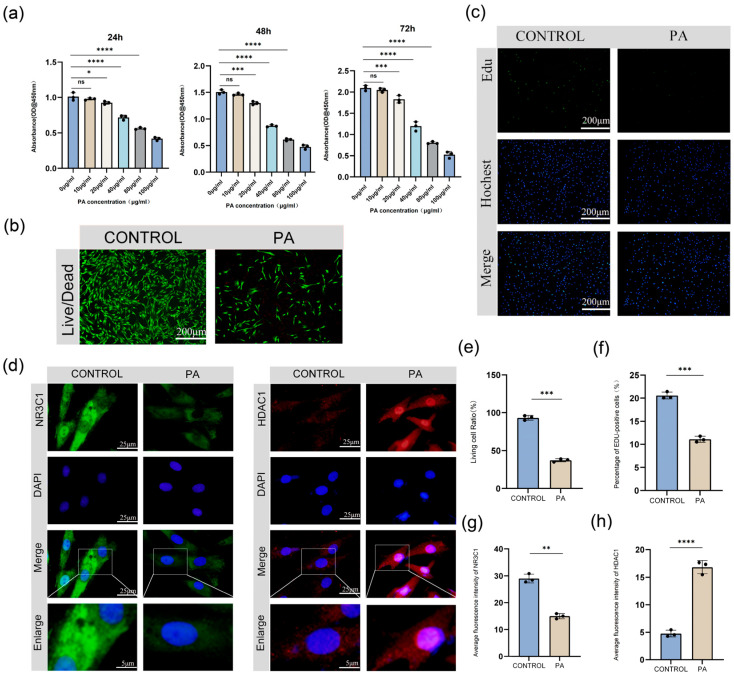
Effects of PA6-MPs on IVDD: (**a**) Concentration-dependent CCK-8 results. (**b**) Live/dead cell staining results (scale bar = 200 μm; green fluorescence indicates live cells). (**c**) EdU staining results (scale bar = 200 μm; green fluorescence indicates EdU-positive proliferating cells, blue fluorescence (Hochest) indicates cell nuclei). (**d**) Immunostaining results for NR3C1 protein expression (scale bar = 25 μm; high magnification: 5 μm; green fluorescence indicates NR3C1 protein, blue fluorescence (DAPI) indicates cell nuclei); Immunostaining results for HDAC1 protein expression (scale bar = 25 μm; high magnification: 5 μm, red fluorescence indicates HDAC1 protein, blue fluorescence (DAPI) indicates cell nuclei). (**e**) Statistical analysis of live/dead cell staining results. (**f**) Statistical analysis of EdU staining results. (**g**) Statistical analysis of NR3C1 protein expression immunostaining results. (**h**) Statistical analysis of HDAC1 protein expression immunostaining results. Data are presented as mean ± standard deviation, *n* ≥ 3; * *p* < 0.05, ** *p* < 0.01, *** *p* < 0.001, **** *p* < 0.0001.

CCK-8 assay results demonstrated that PA6-MPs inhibited NP viability in a concentration-dependent manner. Inhibition of cell viability was observed at a concentration of 40 μg/mL, with a significant inhibitory effect at 80 μg/mL (Figure 6a). Therefore, a concentration of 50 μg/mL PA6-MPs was selected for subsequent intervention experiments. Results from live/dead cell staining assays showed that after 48 h of treatment with 50 μg/mL PA6-MPs, the number of viable cells was significantly reduced compared to the control group (Figure 6b,e). These findings demonstrate that PA6-MPs exert significant cytotoxic effects, markedly reducing NP viability.

EdU staining further confirmed that the number of EdU-positive cells (cells in the proliferative phase) in the PA6-MP-treated group was significantly lower than in the control group (Figure 6c,f), indicating that PA6-MPs can effectively inhibit the proliferative activity of NPs.

IF results revealed that treatment with PA6-MPs significantly downregulated the fluorescence intensity of NR3C1 protein (Figure 6d,g). Concurrently, the localization of NR3C1 within the cell nucleus was markedly reduced, suggesting an inhibition of its intranuclear transcriptional regulatory function. In contrast, PA6-MP treatment led to a significant upregulation of HDAC1 protein fluorescence intensity (Figure 6d,h), accompanied by enhanced nuclear enrichment of HDAC1, indicating an increase in its deacetylase activity and potential involvement in regulating cellular epigenetic modifications. These findings suggest that PA6-MPs may disrupt intranuclear transcriptional regulation and epigenetic modifications—thereby compromising the normal function of NPs—by downregulating NR3C1 expression and activity while upregulating those of HDAC1.

Therefore, this study demonstrates that PA6-MPs participate in the pathological processes of IVDD by inhibiting the viability and proliferation of NPs in a concentration-dependent manner, through the modulation of NR3C1 and HDAC1 expression and nuclear localization. These findings provide an experimental basis for further investigating the potential role of PA6 in IVDD and assessing its associated health risks.

## 4. Discussion

Existing studies have demonstrated that PA6-MPs exhibit a preferential accumulation in human skeletal tissues, particularly in the intervertebral disc (IVD), consistent with previously reported tissue-specific distribution patterns of microplastics [10]. Compared with common MPs such as polystyrene (PS) and polyethylene (PE), PA6-MPs possess stronger protein-binding capacity and tissue affinity due to their polar amide bonds, which may facilitate their retention in hypovascular tissues such as bone and IVD [28]. From an anatomical perspective, the IVD represents a confined microenvironment with limited vascular supply and reduced clearance capacity, resulting in inefficient elimination of exogenous particulate matter. In addition, continuous bone remodeling and cartilage matrix turnover in skeletal tissues may further promote adsorption and retention of foreign particles, including PA6-MPs.

Furthermore, different types of MPs have been reported to induce distinct levels of cytotoxicity, oxidative stress, and inflammatory responses [29]. As a widely used polymer in textiles and daily commodities, PA6-MPs may therefore pose specific biological risks. Current evidence suggests that PA6-MPs may enter the human body via inhalation or ingestion, subsequently distribute systemically through circulation, and ultimately diffuse into the IVD via the cartilaginous endplate. Due to its poor vascularization, low metabolic activity, and limited clearance capacity, the IVD may facilitate particle accumulation, leading to sustained cellular stress and contributing to nucleus pulposus (NP) injury and IVDD progression. Collectively, these findings suggest that PA6-MPs may exert long-term deleterious effects on the IVD microenvironment through direct physicochemical interactions and/or biologically active effects, thereby contributing to degenerative changes.

To gain a comprehensive understanding of the potential toxicity and underlying molecular mechanisms of PA6-MPs in IVDD, this study employed a systematic approach incorporating network toxicology and molecular docking methods. PA6-MPs were selected as the primary subject of investigation. By leveraging the principles of network toxicology and systems biology, and integrating bioinformatics, big data analysis, and multi-omics technologies, we constructed a “compound–toxicity–target” interaction network to elucidate their complex biological mechanisms. Additionally, molecular docking techniques were employed to predict the specific interaction patterns between PA6-MPs and their potential target proteins. To validate these computational predictions and translate them into empirical evidence, we conducted in vitro cellular experiments—including CCK-8 assays, live/dead cell staining, EdU assays, and IF assays—to further explore the impact of PA6-MPs on the initiation and progression of IVDD.

Using a network toxicology approach, we first constructed a PPI network via the STRING database, identifying NR3C1, MAOB, HDAC1, and AKR1B1 as key regulatory factors mediating the effects of PA6-MPs on IVDD. Subsequent GO and KEGG enrichment analyses revealed that these targets converge on critical pathways involved in anti-inflammation and stress homeostasis, thereby may contribute to IVDD progression through mechanisms related to oxidative stress, inflammatory dysregulation, and metabolic disturbances. Molecular docking simulations further confirmed stable intermolecular interactions between PA6-MPs and these core proteins. Finally, in vitro experiments conducted under environmentally relevant exposure conditions validated that PA6-MPs can inhibit the viability and proliferation of NPs in a concentration-dependent manner by modulating the expression and nuclear localization of NR3C1 and HDAC1. These findings provide an experimental basis for further investigating the potential role of PA6 in IVDD and assessing its associated health risks. Collectively, they suggest that PA6-MPs influence IVDD through interconnected mechanisms involving inflammation, apoptosis, and biomechanical alterations, providing preliminary experimental evidence to support a causal link between PA6-MP exposure and the pathogenesis of IVDD.

Network toxicology analysis further identified potential targets involved in the synergistic effects of PA6-MPs and IVDD. A total of 222 putative targets associated with PA6-induced IVDD were identified, and 10 core targets were selected, including NR3C1, MAOB, HDAC1, and AKR1B1. These 10 intersection targets were imported into the STRING database for PPI network analysis, where NR3C1, MAOB, HDAC1, and AKR1B1 were found to occupy central positions. Under normal physiological conditions, these targets are involved in regulating processes such as oxidative stress, inflammatory responses, and metabolic homeostasis. Dysfunction of these targets may contribute to IVDD progression by disrupting intracellular antioxidant defenses, amplifying inflammatory cascades, and interfering with substance and energy metabolism.

Specifically, NR3C1—a glucocorticoid receptor—is a member of the nuclear receptor superfamily and plays a pivotal role in the pathophysiology of IVDD. As a ligand-dependent transcription factor, NR3C1 mediates the anti-inflammatory and immunosuppressive effects of glucocorticoids (such as cortisol) and regulates inflammatory responses through a variety of complex mechanisms [30]. These mechanisms involve the activation and repression of gene expression, regulation of non-coding RNA networks, and modulation of immune cell function. The downregulation of NR3C1 expression or impairment of its function may contribute to the progression of IVDD. When NR3C1 function is compromised, pro-inflammatory pathways, such as the NF-κB pathway, become aberrantly activated, leading to the upregulation of matrix metalloproteinases and a disintegrin and metalloproteinase domain-containing proteins, which exacerbates the degradation of the ECM. Additionally, NR3C1 dysfunction contributes to increased apoptosis of NPs and the release of inflammatory cytokines (such as IL-1β, TNF-α, and IL-6),thereby potentially contributing to the initiation and progression of IVDD. Inflammatory cytokines, particularly IL-1β, play a critical role in advancing IVDD by accelerating NPs senescence and apoptosis, as well as intensifying ECM degradation [31]. Diminished NPs viability is a key hallmark of IVDD. As NPs are the predominant cell type within the IVD nucleus pulposus, their functional impairment can directly contribute to the development of IVDD [32]. Moreover, NR3C1 serves as a pivotal regulator of cellular stress; disruptions to its function can lead to the inactivation of anti-inflammatory pathways, thereby exacerbating chronic inflammation within the IVD [33]. By regulating inflammatory responses and cellular homeostasis at multiple levels, NR3C1—and specifically its dysfunction—represents a key molecular mechanism underlying the pathogenesis and progression of IVDD.

HDAC1 is a class of enzymes that regulate gene expression by removing acetyl groups from histones, thereby influencing gene transcription through modulation of histone acetylation levels [34]. It plays a pivotal role in both the physiological and pathophysiological processes of the human IVD and is particularly closely associated with IVDD.HDAC1 may influence the onset of IVD inflammation by regulating the expression of inflammatory cytokines (e.g., IL-1β and TNF-α). Additionally, as an epigenetic regulator, HDAC1 has been shown to modulate inflammatory signaling within the vascular endothelium [35]. Furthermore, the senescence and apoptosis of NPs represent core features of IVDD, and HDAC1 may exert its influence on NPs by modulating apoptotic pathways (e.g., p53 and Bax/Bcl-2) [36]. Ultimately, HDAC1 dysfunction may alter the gene expression profile associated with IVDD, disrupting the delicate balance between cellular proliferation, differentiation, and apoptosis.

The results of GO and KEGG enrichment analyses provide system-level corroboration for the aforementioned hypothesis. Differentially expressed genes were found to be significantly enriched in biological processes such as the response to ketone bodies, negative regulation of secretion, prostaglandin metabolism, and disruption of cellular structural and functional homeostasis. Furthermore, these genes were enriched in signaling pathways related to chronic inflammation, oxidative stress, stress due to viral infection, neurotransmitter regulation, hematopoiesis, and metabolic disorders. Moreover, prostaglandin metabolism plays a pivotal role in the inflammatory cascade of IVDD. Its upstream arachidonic acid metabolic pathway serves as a critical regulatory node for inflammation, influencing the onset and progression of IVDD [37]. Concurrently, cellular metabolic dysfunction is a significant hallmark of IVDD, specifically manifested as a reduced capacity of NPs to synthesize the ECM [38,39]. Furthermore, although IVDD is not a form of cancer, studies have revealed that certain signaling pathways intimately linked to the pathogenesis and progression of cancer also play a role in IVDD, suggesting the potential existence of shared molecular mechanisms or aberrant signaling activation. Examples include the NF-κB signaling pathway [40] and the PI3K/AKT signaling pathway [41]. These pathways exhibit a high degree of overlap with the biological effects potentially induced by PA6-MPs. This suggests that upon entering the IVD, PA6-MPs may—either directly or indirectly—disrupt the function of core target molecules such as NR3C1, thereby compromising intracellular redox homeostasis and anti-inflammatory homeostasis. This disruption ultimately triggers persistent inflammation and oxidative damage. Under the influence of epigenetic regulatory factors such as HDAC1, relevant degenerative gene expression programs are activated. Coupled with enzymatic metabolic dysregulation—exemplified by AKR1B1—these factors collectively accelerate NP apoptosis and lead to decreased ECM synthesis alongside increased degradation within IVD tissues, ultimately driving structural disruption and functional loss of the IVD, thus potentially contributing to the progression of IVDD [42,43].

Furthermore, molecular docking results indicate that PA6 is capable of spontaneously binding to all four core targets with low binding energies and forming stable molecular conformations. The low binding energies imply that these bindings are thermodynamically feasible, providing important theoretical computational evidence that PA6-MPs have the potential binding capacity to these core proteins and may affect their functions. Notably, molecular docking only serves as a theoretical prediction approach, and further biochemical experimental validations are still required to confirm these interactions.

Although our approach—based on network toxicology and molecular docking—has provided valuable insights into the molecular mechanisms by which PA6-MPs induce IVDD, experimental validation remains crucial. The PA6-MP-mediated suppression of NR3C1 expression and nuclear translocation indicates impaired transcriptional regulation, which subsequently weakens the maintenance of normal physiological activities in nucleus pulposus cells. In contrast, the abnormal elevation of HDAC1 expression and nuclear enrichment suggests disordered epigenetic regulation and enhanced aberrant deacetylation. Collectively, these synergistic alterations at the molecular level ultimately lead to impaired viability and proliferative capacity of nucleus pulposus cells, elucidating the potential mechanism underlying microplastic exposure-induced dysfunction of nucleus pulposus cells at the cellular and molecular levels, and providing experimental evidence for dissecting the pathological mechanism of environmental pollutant-related intervertebral disc degeneration. These in vitro experimental results are highly consistent with our bioinformatics predictions, unequivocally confirming that PA6-MPs can interfere with intranuclear transcriptional regulation and epigenetic modifications within NPs—specifically by modulating the expression and nuclear localization of key target proteins such as NR3C1 and HDAC1—thereby inhibiting NP viability and proliferation in a concentration-dependent manner. This study provides direct experimental evidence regarding the molecular mechanisms underlying PA6-MP-induced IVDD, while also offering critical support for further investigations into the potential role of PA6 in IVDD and for the assessment of its associated health risks.

### Limitations of the Study

Although this study revealed a novel association between PA6-MPs and intervertebral disc degeneration, several limitations should be acknowledged:

(1) Relatively limited experimental system: The absence of in vivo animal studies precluded biological validation of a causal relationship between PA6-MPs and intervertebral disc degeneration. In addition, the use of a single cell type and a limited treatment duration did not address the interactions among multiple cell populations within the intervertebral disc. Furthermore, this study only conducted in vitro direct exposure experiments without modeling physiological exposure routes (e.g., inhalation, gastrointestinal ingestion), which may limit the extrapolation of our findings to real-world environmental scenarios.
(2) Insufficient validation of core targets and molecular mechanisms: Functional validation using siRNA-mediated knockdown or pharmacological inhibition was not performed, and conclusions were therefore primarily correlative. Key indicators related to inflammation, oxidative stress, and apoptosis were not evaluated, and downstream signaling pathways were not further validated by qPCR and Western blot analyses.
(3) Inadequate microplastic characterization and exposure protocols: The physicochemical properties of PA6-MPs (e.g., morphology, particle size, and zeta potential) were not systematically characterized. Only direct in vitro exposure was employed, without simulating physiological uptake or in vivo transport. Moreover, dose- and size-dependent effects were not fully elucidated, and long-term exposure to environmentally relevant, small-sized, low-dose PA6-MPs was not investigated.
(4) Inherent limitations of analytical methods: Network toxicology analyses are constrained by available databases and algorithms, which may introduce bias as well as false-positive and false-negative results. Molecular docking reflects potential binding affinity but cannot determine binding specificity or regulatory function, necessitating further biochemical validation.
(5) Incomplete assessment of particle size and dose effects: Experimental conditions were limited, and the biological differences associated with variations in particle size, concentration gradients, zeta potential, FTIR/Raman spectra, and surface chemical properties were not systematically analyzed.

## 5. Conclusions

This study demonstrates that PA6 microplastics are associated with the progression of IVDD and may represent a previously underrecognized environmental risk factor. Mechanistically, PA6-MPs interact with key regulatory targets, including NR3C1 and HDAC1, thereby disturbing inflammatory and oxidative homeostasis in nucleus pulposus cells and leading to concentration-dependent suppression of cell viability and proliferation. These findings suggest that microplastic exposure may contribute to disc degeneration by perturbing cellular regulatory networks essential for tissue homeostasis.

Collectively, this work provides supportive evidence linking environmental microplastic exposure to IVDD and offers insight into the potential biological basis underlying this association. It also highlights the importance of incorporating environmental factors into the understanding of spinal degenerative diseases and establishes a foundation for future studies focused on exposure-related risk assessment and the development of targeted preventive or therapeutic strategies.

## Figures and Tables

**Table 1:** Key Targets.

Serial Number	Target	Degree Value	Closeness Centrality	Betweenness Centrality
1	NR3C1	6	0.75	0.25
2	MAOB	6	0.75	0.23
3	HDAC1	5	0.69	0.06
4	AKR1B1	4	0.64	0.08
5	PTGER3	4	0.6	0.07
6	CDK4	4	0.6	0.04
7	SIGMAR	4	0.64	0.06
8	CA9	3	0.53	0.01
9	CES2	2	0.5	0
10	PTGER2	2	0.5	0

## Data Availability

Data will be made available on request.
